# An Online Learning Module to Increase Self-Efficacy and Involvement in Care for Patients With Advanced Lung Cancer: Research Protocol

**DOI:** 10.2196/resprot.5547

**Published:** 2016-08-08

**Authors:** Anna Janssen, Tim Shaw, Adnan Nagrial, Christopher Pene, Melanie Rabbets, Matteo Carlino, Clare Zachulski, Jane Phillips, Robert Birnbaum, Tejal Gandhi, Paul Harnett

**Affiliations:** ^1^ Research in Implementation Science and eHealth Faculty of Health Sciences University of Sydney Sydney Australia; ^2^ Crown Princess Mary Cancer Centre Westmead Hospital Sydney Australia; ^3^ Faculty of Health University of Technology Sydney Syndey Australia; ^4^ Partners HealthCare Boston, MA United States; ^5^ National Patient Safety Foundation Boston, MA United States

**Keywords:** patient education, eHealth, online learning, lung cancer, palliative care, side-effect management, patient safety and quality, patient self-efficacy

## Abstract

**Background:**

Improving patient care for individuals with lung cancer is a priority due to the increasing burden of the disease globally. One way this can be done is by improving patient self-management capabilities through increasing their self-efficacy. This can improve positive outcomes for patients with chronic conditions and increase their ability to manage the challenges of such illnesses. Unfortunately, patients with chronic conditions often struggle to travel far from home to engage with patient education events, a common means of improving self-efficacy. The development of more accessible tools for improving patient self-efficacy is required to increase quality of life for patients with chronic conditions.

**Objective:**

To evaluate the feasibility of delivering symptom identification and management information to patients with advanced lung cancer using an online program.

**Methods:**

This article describes a pre-post test study to evaluate a Qstream online learning platform to improve patient self-efficacy for managing advanced lung cancer symptoms. Undertaking this program should increase participant knowledge about the side-effects they may experience as a result of their treatment and in turn increase help-seeking behavior and self-efficacy for the participant cohort. Quantitative data collected by the Qstream platform on the completion rates of participants will be used as a tool to evaluate the intervention. Additionally, validated scales will be used to collect data on patient self-efficacy. Qualitative data will also be collected via an exit survey and thematic content analysis of semi-structured interviews.

**Results:**

The research is in the preliminary stages but thus far a protocol has been approved in support of the project. Additionally, advisory committee members have been identified and initial meetings have been undertaken.

**Conclusions:**

Development of new approaches for increasing patient understanding of their care is important to ensure high quality care continues to be delivered in the clinical setting.

## Introduction

In 2012, 1.82 million people globally were diagnosed with lung cancer, making it the most commonly diagnosed cancer [1]. The World Health Organization predicts that by 2020 that number will increase to 2.2 million [2]. In Australia, nearly 80% of lung cancers are diagnosed at an advanced stage when prognosis is poor, and the overall survival rate is only 15% [3]. These patients often experience multiple symptoms as a result of their disease and/or treatment, including unrelieved pain, breathlessness, distress, and nausea and vomiting [4-5]. Appropriate management of these symptoms is important due to the negative impact on patients, which may include hospitalization if these symptoms are poorly controlled [6].

Managing symptoms in the community requires patients and their caregivers to have good self-management capabilities to ensure they are capable of optimally handling both pharmacological and nonpharmacological interventions. Factors that reduce patient self-efficacy include low health literacy and lack of involvement in treatment decision making. This is particularly concerning considering the literature has shown that both health literacy and involvement in shared decision making significantly impact patient satisfaction and treatment outcomes [7]. There is also evidence that patients with lung cancer may have a diminished understanding of their illness and prognosis and less support in facing end-of-life issues [3]. As a result, these patients are less likely to be proactive in symptom management or demonstrate help-seeking behaviors that could improve their quality of life.

The ability of patients to self-manage aspects of treatment is important because it has been shown to improve outcomes for patients. Doing so can also increase the likelihood patients will successfully manage the challenges of chronic illness [8-9]. One approach to doing this is through the delivery of face-to-face patient education programs. However, these programs could also be delivered online, increasing accessibility for patients unwilling or unable to attend patient education events as a result of their condition. Additionally, there is evidence to suggest an online self-management program can impact self-care and health behaviors in patients [10].

This protocol describes a methodology designed to develop and evaluate a new approach to increasing disease literacy and help-seeking behavior in patients, a high priority for clinicians and patients alike [11]. In addition to the potential benefit of reducing hospital use costs, earlier treatment of side-effects resulting from better patient education may lead to patients receiving more of their planned therapy. A phase I feasibility study design is being used to evaluate the impact of an online learning program on self-efficacy and help-seeking behavior in patients with advanced lung cancer. The project aims to improve the quality of life for these patients by increasing their ability to identify and manage symptoms of their treatment.

## Methods

### Aims

The proposed study evaluates the feasibility of delivering an online self-efficacy program to patients with advanced lung cancer using an online education platform. This program will provide patients with relevant information to identify symptoms resulting from their treatment and provide them with strategies to manage these symptoms when they occur. Additionally, the study aims to assess the impact of the intervention on patient self-efficacy and help-seeking behavior.

In the context of this study, feasibility, including whether the patient population will complete a self-efficacy program delivered online, is being evaluated from the perspective of the patient and not the clinical organization. Additionally, the study will look at whether participants found the program beneficial for identifying and managing their treatment symptoms. This will be evaluated using semistructured interviews.

### Design

A pre-post test design is being used to evaluate an online learning program for improving patient self-efficacy in managing symptoms of treatment for advanced lung cancer. Use of the program should increase participant knowledge about side-effects they may experience and in turn increase help-seeking behavior and self-efficacy for the patient cohort. The online module in this study will be delivered on the Qstream spaced education platform.

Qstream is an evidence-based form of online education that has been shown to improve knowledge acquisition, enhance knowledge retention, and change behavior [12]. Participants in Qstream courses receive repeating, short, case-based questions as well as expert feedback via email in a reinforcing pattern over a number of weeks. The methodology is based on two core psychological research findings: the spacing and testing effects. The *spacing effect* refers to the finding that educational encounters repeated over time increase the acquisition and retention of knowledge. The *testing effect* refers to the finding that the process of testing not only measures knowledge but also improves retention [13-14].

The Qstream spaced learning platform has repeatedly been shown in medical professional development to impact learner knowledge, resulting in behavior change [15]. However, its utility as a tool for engaging with patients has yet to be explored. Existing research on health literacy has shown that improving patients’ knowledge about their treatment can positively impact on quality of life by reducing anxiety and mood disturbances [16]. However, current interventions aimed at improving patient knowledge about treatment side-effects are often either health provider–focused applications or passive dissemination of information in written or Web-based materials [17]. In this study, the Qstream platform will be used for dissemination of key information in a dynamic, personalized, and active manner.

Based on the current research into Qstream as a tool for knowledge dissemination and reinforcement, there is reason to believe it has potential as a means of improving self-efficacy for cancer patients [12]. In addition, the platform has been shown to be easily accessible to learners due to its ability to deliver short, focused pieces of key information at times that suit the learner [18], making the platform adaptable to individual patient information needs.

Quantitative data collected by the Qstream platform on the completion rates of participants will be used as a tool to evaluate the intervention. Additionally, validated scales will be used to collect data on patient self-efficacy. Qualitative data will also be collected via a survey at the conclusion of the module and thematic content analysis of semistructured interviews.

### Setting

This study will be undertaken within the Crown Princess Mary Cancer Centre, Westmead Hospital, Sydney, Australia, which runs a dedicated clinic to support patients with a new diagnosis of lung cancer.

### Study Inclusion and Exclusion Criteria

Eligible participants will be age 18 years or older with a new diagnosis of stage II to stage IV lung cancer and a life expectancy greater than three months. Additionally, participants will need access to an Internet connection and a level of spoken and written English adequate to understand the Qstream cases. Patients who are considered too physically or mentally unwell to participate in the study, do not have adequate English skills, or do not have access to the Internet will be excluded from this study.

A clinical nurse consultant experienced in supporting patients with advanced lung cancer will meet with potential participants to assess their eligibility. This identification process is being used instead of alternative methods such as the Eastern Cooperative Oncology Group (ECOG) status or treatment algorithm due to the difficulty of determining whether patients are physically well enough to participate in the study.

### Study Recruitment

The research team aims to recruit 60 patients from the new lung cancer clinic between January and June 2016. Given that approximately 40 new patients are referred every month, we estimate it will take six months to recruitment 60 patients. This cohort size accounts for an attrition rate of 30%, consistent with other online programs [19].

### Developing the Side-Effect Management Online Learning Module

A brief review of the literature will be undertaken to identify which key symptoms, such as pain management or constipation, are identified as having significant quality-of-life impact for patients with advanced lung cancer. The identified symptoms will inform the focus of the side-effect management program. In addition to a literature review, a local retrospective record audit of between 40 and 60 patients who attended the new lung cancer patient clinic at Westmead Hospital will be undertaken. The local audit will review the symptom burden in the local population to ensure it aligns with the literature.

To oversee the development of the side-effect management program, an expert advisory committee will be convened with representatives from a range of clinical disciplines including at least one representative from the medical oncology, nursing, and palliative care services. This advisory group will have a central role in determining the key messages of the online program, which will be used to develop cases for the Qstream platform. Additionally, the advisory committee will review and prioritize the key domains that the side-effect management program will address.

The Qstream program will consist of approximately one dozen cases in order to convey necessary information. Cases will be developed in two different categories: symptom identification and symptom management, including when to seek medical assistance. Cases will have a consistent format and include an example of a common event encountered as part of lung cancer treatment along with advice on how to address this scenario. In each key symptom domain, there will be a case example guiding participants through identification of a symptom and when and how to seek help if a symptom is unmanageable.

**Figure 1:**
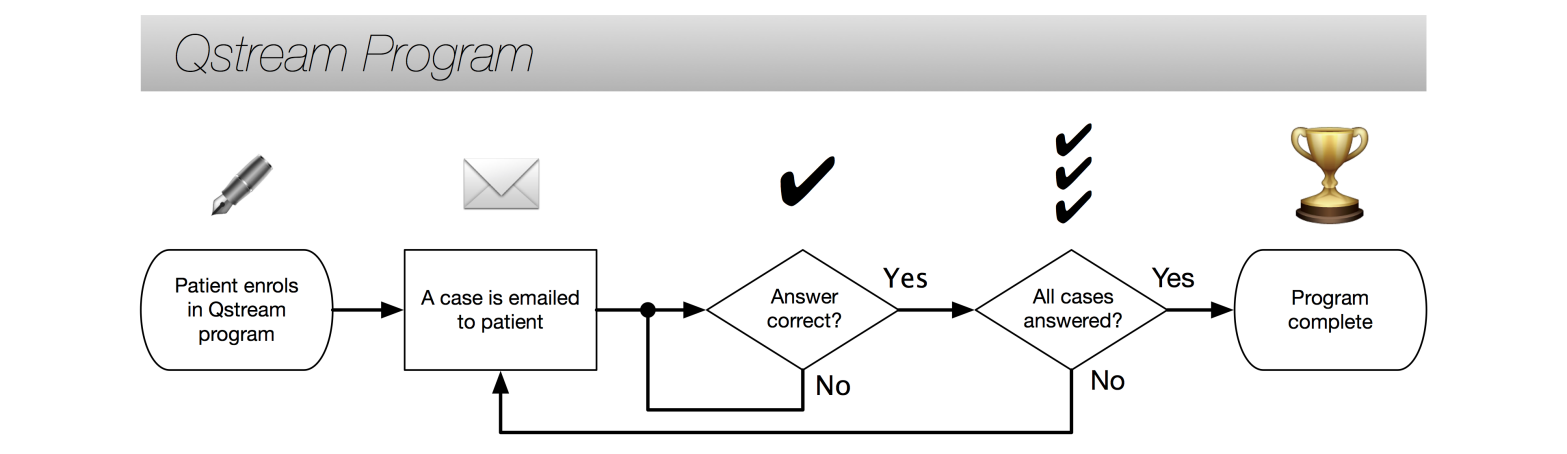
A flow chart showing how patients work through the Qstream program.

For each case, participants will be given a choice of options for how to respond to the situation, of which they will select one. Once participants have selected an option they will be shown how their response compares to other participants undertaking the program. All participants will be given aliases to use in the Qstream platform to ensure patient confidentiality is maintained. In addition to presenting responses of other participants, each case will contain video feedback from a member of the lung cancer team at Westmead Hospital to reinforce the correct approach in a friendly, accessible, and personalized manner. Finally, written feedback will be provided along with links to further resources for participants to explore if they wish. See Figure 1 for a flow chart of patient movement through the Qstream program.

### Implementing the Side-Effect Management Online Learning Module

Once the side-effect management module has been completed it will be made available to study participants. All participants will be automatically enrolled in the Qstream program and receive cases via email or smartphone in the following manner:

1. Each participant will be sent an email every 2 days containing at least 2 questions.

2. If they answer a question incorrectly, it will be re-sent 5 days later.

3. If they answer a question correctly, the question will be re-sent 8 days later.

4. If a question is answered correctly twice in a row, the question will be retired.

5. The course will be completed once all questions have been retired.

We anticipate that most participants will take between 4 and 6 weeks to complete the Qstream program.

### Evaluating the Side-Effect Management Online Learning Module

#### Baseline Data Collection

Baseline data will be collected for the study prior to the dissemination of the Qstream intervention. The research team will use validated surveys to collect information on participant self-efficacy, technical literacy, and involvement in care.

Six weeks after completion of the course, participants will be asked to repeat the survey and answer a number of questions evaluating the Qstream program. Additionally, patients will be invited to participate in an endpoint semistructured interview to evaluate their perception of the impact of the course on their self-efficacy and quality of life. See Table 1 for the tools being used to collect data throughout the study and the time points at which they are being used.

Finally, metrics collected routinely by the Qstream platform will be reviewed to determine usage of the program. A particular focus will be on the number of participants who complete the program and how many cases were completed by participants who do not.

**Table 1:** Overview of measurements used in the study and timing of data collection.

Measures	Pre-intervention	Intervention	Post-intervention
Self-Efficacy for Managing Chronic Disease 6-Item Scale	X		X
Online Technologies Self-Efficacy Scale (OTSES)	X		
Lerman Perceived Involvement in Care Scale (PICS)	X		
Eastern Cooperative Oncology Group (ECOG) score	X		X
Symptom diary		X	
Exit survey			X
Semistructured interviews			X

#### Self-Efficacy for Managing Chronic Disease 6-Item Scale

The Self-Efficacy for Managing Chronic Disease 6-Item Scale [20], developed by the Stanford Patient Education Research Center, will be used to rate participants across several domains common in chronic disease including emotional functioning and symptom control. The scale is based on a larger self-efficacy scale developed for the Chronic Disease Self-Management Study [21].

The scale requires respondents to rate 6 items on a scale of 1 to 10, where 1 indicates “not at all confident” and 10 indicates “totally confident.” The scale was tested on a cohort of 244 individuals with chronic disease: the mean score was 30.24, with a standard deviation of 6.28 and internal consistency reliability (Cronbach alpha) of .93 [20]. Although the tool has not been used with lung cancer patients, it has been used to measure self-efficacy in breast cancer patients [22].

#### Online Technologies Self-Efficacy Scale

To evaluate the technical literacy of participants, the Online Technologies Self-Efficacy Scale will be used. The validated scale was developed in 2000 to assess respondent confidence across a range of domains including use of email, use of Web browsers, and navigation of websites [23]. The scale was tested on a cohort of 330 tertiary level students. The Cronbach alpha is .95.

#### Lerman Perceived Involvement in Care Scale

The final validated scale being used is the Lerman Perceived Involvement in Care Scale (PICS). This tool will be used to collect baseline data on participant perception of involvement in their care. The PICS has 13 items that were validated using a test cohort of 131 individuals. The Cronbach alpha is .73. An alpha coefficient of .60 was determined using an independent sample of 81 patients [24]. This tool has been used effectively with a number of patient populations including a group of 1081 early stage breast cancer patients [25].

#### Eastern Cooperative Oncology Group Score

The ECOG score will used to collect baseline data regarding disease progression and other treatment information. Information for this ranking, along with basic demographic data such as gender, age, and postal code, will be obtained from the patient medical record.

#### Symptom Diary

To minimize recall error, participants will be asked to keep a diary to capture incidences of symptoms that occur during the intervention [26]. Study participants will be provided with a prestructured diary booklet to use as a guideline that includes instructions on what to include in entries and an example of an entry. The booklet will be accompanied by a cover letter reenforcing the outline and the disease-specific information to be included. Contact information will be provided for participants who have questions. At the conclusion of the study period, the diary will be evaluated for feasibility as a measurement tool and influence of the intervention on symptom management. The diary entries may be discussed with patients in a later interview.

### Data Analysis

The primary goal of this study is to assess the feasibility of delivering treatment self-management information to patients using the Qstream platform. Quantitative data collected by the platform will be analyzed to gain insight into the uptake and completion rates of the program by the study participants. Additionally, qualitative data will be analyzed to measure the impact of the program on symptom management knowledge, self-efficacy, and help-seeking behaviour.

Quantitative data on patient self-efficacy is being collected via a validated survey administered at two time points: two weeks prior to intervention and two weeks postintervention. All data analysis will be performed using SPSS statistical analysis software (IBM Corp). The after minus before difference will be measured for each patient who completes both the pre- and postsurvey. The average of the before/after difference will be determined to test the hypothesis that the difference is not zero. A matched *t* test will be used at a level of significance set at 0.05. The difference (as a 95% confidence interval) and the *P* value will be presented.

Qualitative data is being collected through semistructured interviews with participants. This data will be thematically analyzed to identify emergent and overarching themes with characteristic quotes used to exemplify each theme. Two researchers will read each transcript and independently create a draft coding scheme. This will be discussed, and any differences will be resolved in consultation with the wider research team. Additional transcripts will be read with iterative refinement of the draft coding scheme. An appropriate software program will be used to organize the data and allow development of themes, subthemes, and higher order themes, with characteristic quotes. The final thematic summary will be sent to a subset of participants who will be phoned for feedback on the accuracy of this summary.

### Ethical and Legal Considerations

As of January 2016, the protocol has been granted ethical approval by the Western Sydney Local Health District Human Ethics Committee. To obtain ethical approval this study was evaluated for compliance with the National Statement on Ethical Conduct in Human Research 2007 and the National Health and Medical Research Council guidelines under Section 95 of the Privacy Act 1988.

### Data Storage Procedure

All data collected from participants will be deidentified and coded with a participant identification code on a master log sheet by a member of the research team. Participant codes will be kept in a password-protected Excel spreadsheet on a password-protected electronic database. Only deidentified information will be used by members of the research team when data analysis is being undertaken.

Information collected for, used in, or generated by this project will remain securely stored in the offices of the principle investigator. For digital information, this will involve storage password-protected personal computers. Paper copies will have identifiers removed and will be stored in a locked filing cabinet.

### Potential Risks

There is a risk that some participants may experience distress during this study. Participants will be provided access to a counseling service if they are experiencing distress. Information on how to contact the counseling service will be provided to participants when they register for the study. Loss of confidentiality is an additional risk of this study. Researchers are minimizing this risk by following the data storage procedure outlined earlier in this protocol.

## Results

This protocol describes a research study in its preliminary stages and, as such, only modest results can be reported. One significant outcome is the development of a comprehensive method for evaluating the impact of a online program for delivering symptom identification and management information to patients.

An expert advisory committee including medical oncologists, palliative care specialists, and clinical nurse consultants has been identified and convened to guide the direction of this project. Initial consultation with the advisory committee has identified symptoms that will be targeted by the Qstream program. The advisory committee has decided pain management, breathlessness, constipation, nausea, and lethargy would be the most relevant areas to address for the patient population in this study. All of these symptoms are identified in the literature as being challenging for patients to self-manage.

## Discussion

### Prinicipal Findings

The development of tools to support patient self-efficacy in managing advanced lung cancer is important due to the significant burden cancer represents globally [1]. Although interventions exist to improve patient health literacy and increase patient involvement in their care, these interventions largely focus on passive dissemination of information [17]. This research protocol explores an online knowledge dissemination module that has been demonstrated to impact both knowledge and behavior [12]. The Qstream platform has not previously been used for patient education but has been used extensively for delivering health education to other groups [15]. As a result, the findings of this study contribute to addressing a clear gap in the current literature.

Although the program is being evaluated in the context of lung cancer, it has potential to be used across a range of other cancers and chronic conditions. The literature on symptom prevalence for patients with advanced lung cancers shows that patients with this condition experience nausea and vomiting, and symptoms resulting in patient hospital admission include pain and dyspnea [6]. These symptoms are not unique to lung cancer and are prevalent across chronic conditions including heart disease, AIDS, and renal disease [27]. If the intervention is effective with patients with lung cancer it may be applicable across a much broader patient population.

Finally, in order to ensure a high quality of care continues to be delivered to patients, the evaluation of new techniques for dissemination of patient information is a priority. Unfortunately the literature to date suggests that there is a gap in knowledge relating to the identification and uptake of appropriate behavior change techniques to promote such behaviors in patients [28]. By evaluating the impact of Qstream on patient self-efficacy and help-seeking behavior we anticipate that a significant contribution will be made to address this gap in the literature.

### Conclusions

Developing and evaluating new techniques for dissemination of patient information to enhance patient self-efficacy and understanding of their care is integral for ensuring high quality care continues to be delivered in the clinical setting. This is particularly important for treatment of chronic conditions such as advanced lung cancer, because optimal delivery of such information can improve patient self-efficacy and ensure a high quality of life is maintained during end-of-life care.

## Abbreviations

ECOG: Eastern Cooperative Oncology Group
PICS: Perceived Involvement in Care Scale
